# Magnitude and associated factors of neonatal mortality among neonates admitted at Dessie comprehensive specialized hospital, Northeast, Ethiopia

**DOI:** 10.3389/fped.2025.1466599

**Published:** 2025-07-10

**Authors:** Berhanu Adugna, Robel Asaminew, Getye Solomon Maru, Haimanot Ayele Eshetie, Endris Mohammed, Ayele Samuel, Abdulmelik Mekonnnen, Nurye Fentaw, Brhanu Belay, Adem Mekonen, Mengstie Nigussie, Fekadeselassie Belege Getaneh, Alemu Gedefie

**Affiliations:** ^1^ Dessie Comprehensive Specialized Hospital, Dessie, Ethiopia; ^2^ Department of Pediatrics and Child Health Nursing, School of Nursing and Mid-Wifery, College of Medicine and Health Sciences, Wollo University, Dessie, Ethiopia; ^3^ Department of Medical Laboratory Sciences, College of Medicine and Health Sciences, Wollo University, Dessie, Ethiopia

**Keywords:** associated factors, Ethiopia, magnitude, neonate, neonatal intensive care unit, neonatal mortality

## Abstract

**Background:**

Neonatal mortality rate is high in sub-Saharan Africa than high-income countries in relation to the growing wealth disparity. Different factors are linked with neonatal mortality in Ethiopia. Identification of the causes of death is the first step in reducing mortality rates. Thus, the aim of this study was to assess the magnitude of neonatal mortality and associated factors among neonates admitted in Neonatal Intensive Care Unit (NICU) in Dessie Comprehensive Specialized Hospital, Northeast Ethiopia.

**Methods:**

A retrospective cohort study was conducted among 1,598 neonates admitted in Neonatal intensive care unit of Dessie Comprehensive Specialized Hospital from 28/06/2022 to 30/03/2023. Demographic and clinical data were abstracted from admission/discharge registration books; perinatal facility-based data abstraction form and patient medical records using data extraction checklist. Bivariable and multivariable analyses were conducted to determine the factors associated with neonatal mortality and variables with an adjusted relative risk (ARR) and its *P*-value < 0.05 were considered statistically significant. Model fitness was computed using Hosme-Lemeshow Goodness of fitness (*P* = 0.847).

**Results:**

Among 1,598 neonates who were admitted in NICU were included in this study of which 914 (57.2%) were males. The magnitude of neonatal mortality was 10.2%. Prematurity (ARR = 2.58, 95% CI: 1.39–4.87, *P* < 0.009), sepsis (ARR = 1.47 95% CI: 1.02–2.11, *P* < 0.036), birth asphyxia (3.59 = 4.36, 95% CI: 2.40–6.87, *P* < 0.008), and respiratory distress syndrome (ARR = 2.93, CI: 1.47–5.30, *P* = 0.011) were independent predictors of neonatal mortality.

**Conclusion:**

The magnitude of neonatal mortality was 10.2% which alarms the need of immediate collaborative actions for reduction of the burden particularly tackling on the causal factors such as prematurity, sepsis and birth asphyxia which leads adverse birth outcomes. Therefore, maternal counseling, giving focused ante natal care as well as behavior change communications might be considered to promote positive behaviors are recommended to avoid the leading causes of neonatal mortality.

## 1 Introduction

Neonatal mortality, which is defined as death within the first 28 days of life, is a crucial indicator for the health and wellbeing of newborns and is increasingly playing a significant role in the total under-five mortality rate. As a result, health officials are paying special attention to neonatal deaths (1). It can be further classified into late newborn fatalities (7–27 days) and early neonatal deaths (0–6 days), which occur after the seventh day but before the 28th day of life (2). The neonatal period, which lasts for the first 28 days of a neonate's existence, is the most dangerous stage of life because a baby is especially prone to making many of the physiological changes required for life outside the uterus at this time (3). The neonatal period is associated with an average global rate of 18 deaths per 1,000 live births (4) and 3–4 million global deaths per year where 99% of them was in low to middle income countries (5).

Despite a continuous decline in under-five mortality, approximately 7,000 newborns die each day, according to a 2017 WHO report (6, 7). However, the global neonatal mortality has significantly decreased from 5 million in 1990 to 2.4 million in 2019, newborn death still accounts for 47% of all deaths among children under the age of 5, with nearly one-third dying on the day of birth and nearly three-quarters dying within the first week of life (8). Furthermore, the evidence from Inter-Agency Group for Child Mortality Estimation reported that around 30 million neonates will die before their fifth birthday between 2017 and 2030 (9).

The burden of neonatal mortality is high in low- and middle-income countries (LMICs) than high-income countries. sub-Saharan Africa and Asia have the highest mortality rates. In the WHO Regions of Africa (28%) and South-East Asia (36%), neonatal mortality rates are two thirds higher (10). From sub-Saharan African countries, Ethiopia had the fifth-highest rate of neonatal deaths in 2016 (90/1,000) (11). Nearly 1 and 2 million deaths occur on the day of delivery and in the first week of life, respectively, and neonatal deaths account for 40% of all mortality of children under the age of 5 (12). When compared to the remainder of the month, where 50% of deaths occur and 75% occur throughout the first 7 days of life, a newborn has a 500 times higher chance of dying within the first 24 h (13). The rate of neonatal mortality is intrinsically linked to the health of the mother and the care she receives before, during and immediately after giving birth. Asphyxia and birth injuries usually result from poorly managed labor and delivery and lack of access to obstetric services (14, 15).

Preterm, prenatal asphyxia, and infections like sepsis, meningitis, and pneumonia account for more than 80% of newborn deaths (16). Particularly in Ethiopia, preterm birth, intrapartum problems, and infections account for roughly 90% of all fatalities. For 31% of neonatal deaths in Ethiopia, neonatal infection is the major contributing factor (17). Neonatal illnesses are challenging to treat with a single therapeutics or clinical interventions but it requires a systematic approach (16). Moreover, offering a continuum of care from pregnancy through birth and the immediate postpartum period, immediate causal factors could be readily avoided (18, 19). In addition, reduced newborn morbidity and mortality can be achieved through skilled care during pregnancy, labour, and the postpartum period (20).

The number of deaths and the percentage of neonatal deaths are predicted to increase to 69 million deaths and 52%, respectively, by 2030. Target 3.2 of the Sustainable Development Goals (SDGs) 3 shifts the focus to newborn health, with the goal of bringing the rate of neonatal mortality below 12 per 1,000 live births by 2030 (21, 22). But, on the basis of current trends more than 60 nations will fall short of SDG target by 2030. Similarly, by 2050 over half of them won't have accomplished the goal (23). Furthermore, the Mini-Ethiopian Demographic and Health Survey (EDHS) report states that Ethiopia has a neonatal mortality rate of 30% per 1,000 live births (4). Neonatal mortality increased from 28 per 1,000 live births in 2016 to 30 per 1,000 live births in 2019 according to a report from EDHS-2019 indicating NMR is still a big public health problem in Ethiopia (24).

Despite a significant reduction in maternal and child mortality in Ethiopia, neonatal deaths are still occurring in substantial numbers. A piece of evidence has also tried to indicate the problem of NMR, but the problem is quite different due to ecological factors. Additionally, previous studies were performed using a small sample size, which might lack generalizability. Thus, evidence with a cohort study design and higher sample size can provide more reliable evidence for scientific communities, as well as policy makers and decision makers. Therefore, the aim of this study was to assess the magnitude of neonatal mortality and to identify associated factors among neonates admitted in Neonatal intensive care unit in Dessie Comprehensive Specialized Hospital, Northeast Ethiopia.

## 2 Methods and materials

### 2.1 Study area, design, period and population

A retrospective cohort study of neonatal death was conducted from 28/06/2022 to 30/03/2023 among a total of 1,598 neonates admitted in Neonatal intensive care unit (NICU) of Dessie Comprehensive Specialized Hospital (DCSH) (Supplementary Data Sheet 1). The DCSH neonatal care unit has a 38-bed capacity with sections of term, preterm and septic/postop sections and equipped with 04 incubators, 07 phototherapy, 13 radiant warmers, 04 CPAP machine, 04 concentrators and 01 pulse oximetry. The unit is headed by senior consultant pediatricians together with a team of 4 general practitioners, interns, 11 neonatal nurses, 12 BSc nurse and other paramedical staff. Chart review of this study was carried-out from 01/05/2023 to 30/05/2023. Neonates chart with missing data for socio-demographic, clinical and epidemiological variables were excluded from the study analysis.

#### 2.1.1 Eligibility criteria

**Figure 1:**
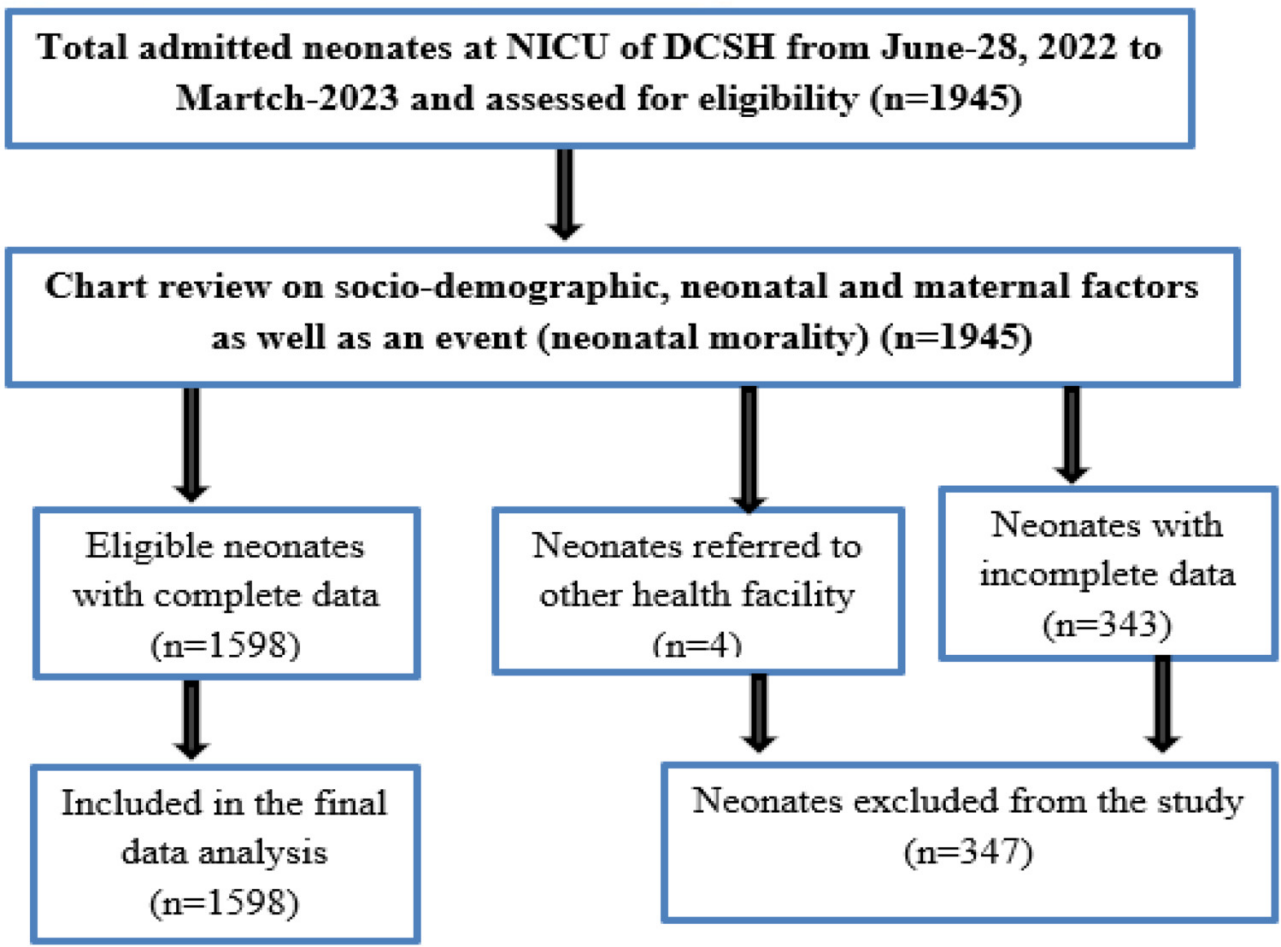
Enrolment procedure of admitted neonates in the study.

All neonates who were admitted in NICU of Dessie Compressive Specialized hospital from June-28, 2022 to March-2023 were included. However, neonates who were referred to other health facilities and those had incomplete medical records were excluded from the study (Figure 1).

#### 2.1.2 Variables

The dependent variable was neonatal mortality while socio-demographic factors (age of the neonate in days and the mother, sex of the neonates, and place of residence), neonatal factors (birth weight, medical illnesses, danger signs, length of stay on admission and body temperature) and maternal factors (ANC service, feeding type to their neonate, breast feeding initiation time, place of delivery, mode of delivery, birth order, gravidity, history of neonatal death, HIV status of the mother, chronic disease of the mother, multiple pregnancies and gestational age) were considered as explanatory variables.

#### 2.1.3 Operational definitions

• **Neonatal death:** Death of a neonate within the first 28 completed days of life that was born alive
• **Early neonatal death:** The death of neonate that occur with in the first 7 days of life
• **Late neonatal death:** Death of a neonate that occur between 7 and 28 completed days of life
• **Morbidity**: is defined as the presence of confirmed medical conditions in a neonate within the first 28 days of life such as sepsis, asphyxia, prematurity, respiratory distress disorder, jaundice, meconium aspiration, congenital malformation, hypoglycemia, low birth weight, hypothermia and anemia etc.

#### 2.1.4 Data collection

Data were abstracted during June-2022–March,2023 from the medical records of the neonates, NICU register book, and Facility based data abstraction form of Perinatal Death Surveillance and Response (PDSR) retrospectively using pre-prepared and pre-tested data extraction checklist containing socio-demographic factors (age of the neonate in date and the mother, sex of the neonates, and place of residence), neonatal factors (birth weight, medical illnesses, danger signs, length of stay on admission and body temperature), and maternal factors of neonatal deaths (ANC service, feeding type to their neonate, breast feeding initiation time, place of delivery, mode of delivery, birth order, gravidity, history of neonatal death, HIV status of the mother, chronic disease of the mother, multiple pregnancies and gestational age). The data was collected by 4 health care workers (1 neonatal nurse and 3 BSc nurse) with a close supervision by senior Maternal neonatal and child health (MNCH) supervisor who had an experience in NICU. Only one person did the data checking and recording per neonate.

#### 2.1.5 Statistical analysis

Patient data were registered into Microsoft excel and analyzed using SPSS version 25 software (SPSS Inc., Chicago, IL, USA). Categorical variables were summarized by frequencies and percentages, and continuous variables were summarized by mean ± standard deviation (SD). Bivariable logistic regression analyses were performed to assess predicting factors associated with neonatal mortality. Variables that were found to be significant in bivariable analysis (*P* < 0.25) were included in the multivariable backward stepwise logistic regression model to identify factors independently associated with neonatal mortality. Model fitness was computed using Hosme-Lemeshow Goodness of fitness (*P* = 0.847). Finally, variables with an adjusted relative risk (ARR) with a *P*-values < 0.05 were considered significant.

## 3 Result

### 3.1 Socio demographic, neonatal and maternal characteristics

A total of 1,598 neonates who were admitted in NICU had complete medical records and included in the study. Majority of the neonates were male (57.2%). Almost tenfold of the neonates were admitted in the age of 1–7 days with the maximum age of 20 days and the median age of 1 day. Around two thirds of (65.7%) of the neonates were from rural residence. Furthermore, around 2/3rd (67.3%) of neonates were delivered from mothers of gestational age 37–42 weeks. Regarding maternal and neonatal factors, nearly half (47.9%) of the neonates were delivered from mothers had history of four and above visits of ANC. With regard to place of delivery, majority 1,085 (67.9%) were delivered at same heath facility. Similarly, majority of neonates 1,309 (81.9%) were also delivered by SVD and 1,594 (99.7%) were single on births. However, more than half of (60.6%) neonates were delivered from mothers with history of 2–4 parity (Table 1).

**Table 1:** Socio demographic, neonatal and maternal characteristics of neonates from June 2022 to March 2023 at Dessie comprehensive specialized hospital.

Variables	Category	Frequency	Percent
Age in days	1–7	1,453	90.9
	8–27	145	9.1
Sex	Male	914	57.2
	Female	684	42.8
Resident	Urban	533	33.3
	Rural	1,065	65.7
Weight (gram)	<1,500	195	12.2
	1,500–2,499	424	26.5
	2,500–3,499	948	59.4
	>=400	31	1.9
Maternal age in years	<=20	78	4.9
	21–34	1,231	77
	>=35	289	18.1
Gestational age	<37	497	31.1
	37–42	1,075	67.3
	>42	26	1.6
Place of delivery	Referred from other health facility	502	31.4
	Home	11	0.7
	Same facility	1,085	67.9
Mode of delivery	SVD	1,309	81.9
	CS	237	14.8
	Instrumental	52	3.3
ANC visit	1–3	832	52.1
	4–8	766	47.9
Multiple pregnancy	Yes	4	0.3
	No	1,594	99.7
Parity	1	470	29.4
	2–4	968	60.6
	≥5	160	10

### 3.2 Magnitude of neonatal mortality

**Figure 2:**
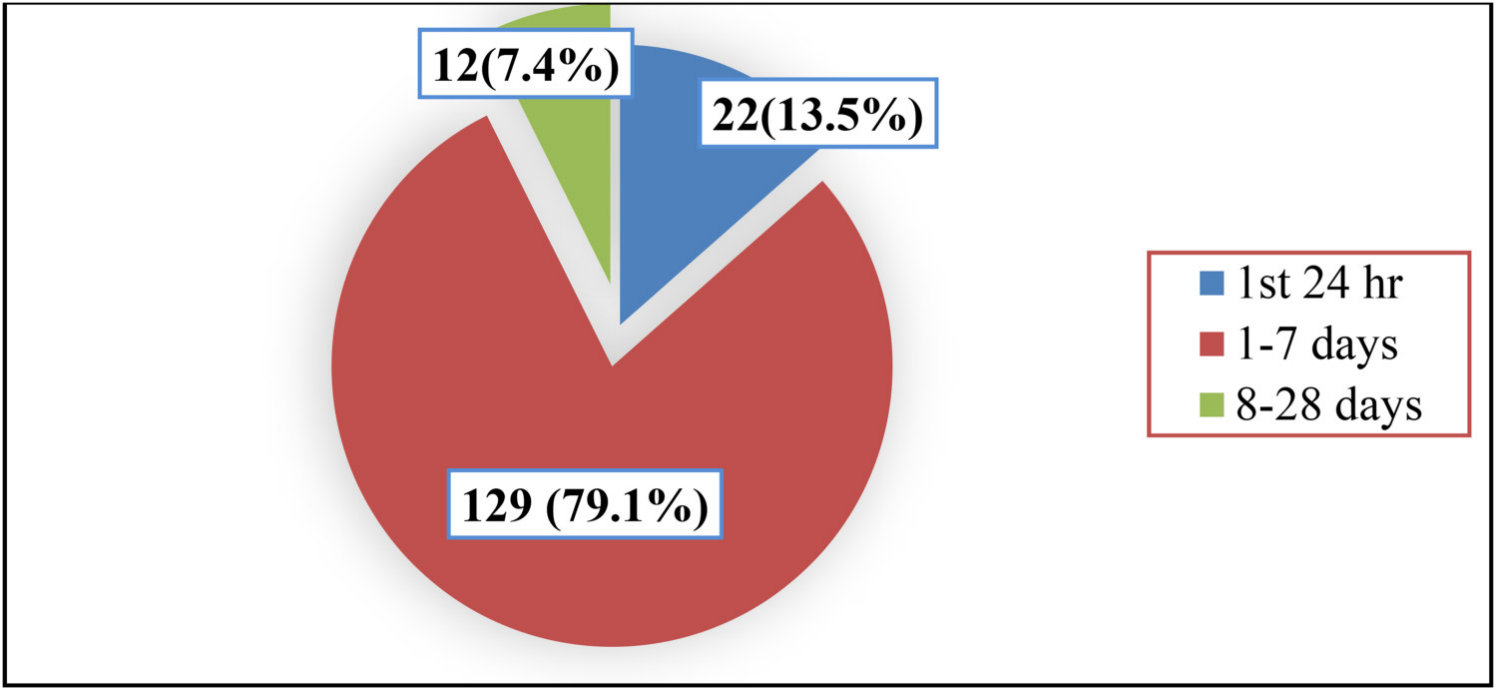
Neonatal deaths according to neonates age categories.

Out of the total 1,598 neonates, 1,435 were improved and discharged alive; however, 10.2% (*n* = 163; 95%CI: 8.8–11.7) of the neonates were died. About 79.1% (129/163) of neonates were died within 1–7 days of birth while 13.5% (22/163) were died within the first 24 h. Totally, the magnitude of neonatal mortality within the first week of birth was nearly 93% (151/163) (Figure 2).

The top three predominant causes of neonatal morbidity or causes of neonatal admission were sepsis (35.2%, asphyxia (19%), and prematurity (18.2%). Likewise, the predominant causes of morbidity were also reported as a causes of neonatal mortality (Table 2).

**Table 2:** Morbidity and mortality status neonates based on their underlying clinical conditions.

S.no	Underlying clinical conditions	Morbidity (*N* = 1,598), No %	Mortality (*N* = 163), No %
1	Sepsis	563 (35.23)	46 (28.2)
2	Asphyxia	303 (19.0)	42 (25.8)
3	Prematurity	292 (18.3)	37 (22.7)
4	Respiratory distress disorder	96 (6.0)	11 (6.8)
5	Jaundice	89 (5.6)	10 (6.1)
6	Meconium aspiration	54 (3.4)	8 (4.9)
7	Congenital malformation	229 (14.3)	22 (13.5)
8	Hypoglycemia	55 (3.4)	5 (3.1)
9	Low birth weight	164 (10.3)	14 (8.6)
10	Hypothermia	205 (12.8)	25 (15.3)
11	Anemia	79 (4.9)	6 (3.7)

**Table 3:** Bivariable and multivariable analysis of risk factors associated with neonatal mortality among admitted neonates in NICU at Dessie comprehensive specialized hospital June 2022–March, 2023.

Characteristics		Outcome		CRR (95%CI)	*P*-value	ARR (95%CI)	*P*-value
		Dead *n* (%)	Alive *n* (%)				
Neonatal age	1–7	151 (10.4)	1,302 (89.6)	0.778 (0.421–1.438)	0.423		
	8–27	12 (8.3)	133 (91.7)	1			
Sex	Female	78 (11.4)	606 (88.6)	1			
	Male	85 (9.3)	829 (80.7)	1.255 (0.907–1.737)	0.170	1.280 (0.922–1.778)	0.140
Mother's age	<20 years	9 (11.5)	69 (88.5)	1			
	20–34 years	124 (10.1)	1,107 (89.9)	1.164 (0.567–2.390)	0.678		
	>=35 years	30 (10.4)	259 (89.6)	1.126 (0.511–2.483)	0.769		
Parental address	Rural	85 (9.6)	798 (90.4)	0.870 (0.629–1.203)	0.400		
	Urban	78 (10.9)	637 (89.1)	1			
ANC visit	1–3 visit	87 (10.5)	745 (89.5)	0.943 (0.683–1.305)	0.724		
	4–8 visit	76 (9.9)	690 (90.1)	1			
Multiple pregnancy	No	47 (10)	423 (90)	0.987 (0.738–1.412)	0.652		
	Yes	116 (10.3)	1,012 (89.7)	1			
Number of parity	1	47 (10)	423 (90)	1.15 (0.907–2.166)	0.129	1.122 (0.464–1.123)	0.148
	2–4	102 (10.5)	866 (89.5)	1.22 (1.01–2.223)	0.118	1.101 (0.985–1.897)	0.132
	>4	14 (8.8)	146 (91.2)	1			
Mode of delivery	SVD	134 (10.2)	1,175 (89.8)	1			
	CS	27 (11.4)	210 (88.6)	0.887 (0.572–1.3,760	0.592		
	Instrumental delivery	2 (10.2)	50 (89.8)	2.851 (0.686–11.850)	0.149		
Prematurity of neonates	No	126 (9.4)	1,210 (90.6)	1			
	Yes	37 (14.1)	255 (85.9)	1.579 (1.066–2.340)	0.023*	2.586 (1.392–4.877)	0.009**
Sepsis	No	77 (7.4)	958 (92.6)	1			
	Yes	86 (15.3)	517 (84.7)	2.082 (1.002–4.048)	0.029*	1.470 (1.025–2.110)	0.036**
RDS	No	146 (9.8)	1,350 (90.2)	1			
	Yes	11 (16.7)	85 (83.3)	1.541 (0.313–0.935)	0.028*	2.932 (1.475–5.301)	0.011**
Asphyxia	No	121 (9.3)	1,174 (90.7)	1			
	Yes	42 (13.9)	261 (86.1)	1.561 (1.072–2.273)	0.020*	3.595 (2.405–6.874)	0.008**
Congenital malformation	No	141 (10.3)	1,228 (89.7)	1			
	Yes	22 (9.6)	207 (90.4)	0.926 (0.577–1.485)	0.749		
Hypothermia	No	138 (9.9)	1,255 (90.1)	1			
	Yes	25 (12.2)	180 (88.8)	0.792 (0.503–1.246)	0.313		
Meconium aspiration	No	155 (10)	1,389 (90)	1			
	Yes	8 (14.8)	46 (85.2)	1.558 (0.722–3.362)	0.258		
Jaundice	No	153 (10.1)	1,356 (89.9)	1			
	Yes	10 (11.2)	79 (88.8)	1.122 (0.569–2.212)	0.740		
Apgar score	<=6	15 (6.9)	201 (93.1)	1.602 (0.926–2.790)	0.092	1.564 (1.897–2.725)	0.115
	>=7	148 (10.7)	1,234 (89.3)	1			
Body temperature	<36.4	131 (10.6)	1,101 (89.4)	0.733 (0.420–1.280)	0.275		
	36.4–37.2	17 (9.5)	162 (90.5)	0.831 (90.402–1.719)	0.618		
	>37.2	15 (8)	172 (92)	1			
Anemia	No	157 (10.3)	1,367 (89.7)	1			
	Yes	6 (7.6)	73 (92.4)	0.713 (0.305–1.666)	0.435		
Average length of stay in NICU	<7 days	142 (10.5)	1,206 (89.5)	1			
	>=7 days	21 (8.4)	229 (91.6)	1.2,849 (0.795–2.074)	0.307		

* indicates the *P*-value of CRR less than 0.05 while **indicates the *P*-value of ARR less than 0.05.

### 3.3 Factors associated with neonatal death among neonates admitted at NICU

The magnitude of neonatal mortality was assessed in the current study using a variety of risk factors. In bivariable and multivariable analysis, Preterm new borns (prematurity of neonates) who had higher risk of death than those who born term babies (ARR = 2.586, 95%CI 1.392–4.877, *P* = 0.009), and those newborn babies who had sepsis with sepsis developed new borns were 1.470 times at risk to die than those non sepsis developed babies (ARR = 1.470, 1.025–2.110, *P* = 0.036). Neonates faced birth asphyxia were 3.595 times at risk to die than those who were non asphyxiated newborns had non asphyxia new borns (ARR = 3.595, CI: 2.405–6.874, *P* = 0.008). Moreover, the risk of neonatal death was about 2.9 times higher in neonates who had respiratory distress syndrome (ARR = 2.932, CI: 1.475–5.301, *P* = 0.011) than their counter parts (Table 3).

## 4 Discussion

Neonatal mortality remains a severe public health issue, especially in low- and middle-income countries like Ethiopia, despite numerous advancements and treatments made to increase the survival of babies. Thus, the aim of the current study was to identify magnitude and associated factors of neonatal mortality at Dessie Comprehensive Specialized Hospital, Northern Ethiopia. The magnitude of neonatal mortality among NICU admitted neonates was 10.2% (95% CI: 8.8–11.7) which was comparable with previous findings reported from 11.4% prevalence in Ethiopia (25). On the other hand, the finding of the current study was lower than the finding of previous Ethiopian studies reported (16.7%) (13) and (18.34%) (26).

Moreover, it was higher than 4.37% neonatal death reported from Ethiopia (27). The variations might be due to study setting such as this study was conducted among neonates in NICU who have high risk of death than their counterparts. In addition, the variation can be explained by differences in skill of health professionals, presence of sociocultural and socioeconomic differences across regions of Ethiopia as well as the difference in sample sizes (9, 28) and economic disparities among study participants (9, 29, 30). Moreover, difference in health seeking behavior and health service utilization such as institutional delivery by skilled care providers and health seeking for sick neonates. As the quality of care can be affected by the availability of equipment and skilled professionals, the higher magnitude of neonatal death found in this study can be claimed to be explained by this condition.

The magnitude of early neonatal death in this study was 93% which was consistent with previous finding reported from northeastern Ethiopia. This is in line with other prior studies done in Diredewa (30) and Mizan- Tapi (9). However, the finding was higher than studies in Jimma and Bahir Dar (15, 31). The possible reason of higher magnitude of early neonatal death could be due to the fact that the first few hours of age after birth are very critical to adapt the environment, simple and inexpensive measures can decrease neonatal mortality in the first 24 h of life. Likewise, high number of prematurity-related deaths (majority of which are secondary to respiratory distress syndrome), where maximum deaths occurred in the first 72 h of age. In addition, it could be due to majority of neonatal mortality in developing countries are related to conditions of labor, intra- partum and the immediate newborn care practices which could be further associated with prematurity, sepsis, respiratory distress syndrome and birth asphyxia. This again clearly pointed out that neonatal survival interventions have to target the intra-partum as well as immediate and early neonatal periods.

The risk of neonatal mortality was significantly associated with clinical characteristics of neonatal factors which aggravates neonatal mortality. In our study, the risk of neonatal mortality was observed to rise as complication of prematurity advances, it was significantly associated with the magnitude of neonatal death (*P* < 0.009). Of 18.3% of the study subjects who were complication of prematurity, 14.5% of neonates were dead, this is due to the fact that, premature neonates are at higher risk of dying due to complications such as respiratory problems, feeding difficulties, poor regulation of body temperature and recurrent infections (32).

Neonates admitted to neonatal intensive care unit with birth asphyxia had 3.595 time at risk of dying than their counterpart. This could be due to the fact that oxygen deficit at delivery (birth asphyxia) can lead to severe hypoxic ischemic organ damage in newborns followed by a fatal outcome or severe life-long pathologies. This finding is consistent with a study done in Somalia region (33). This difference might be due to health service utilization, study period and sample size variations. In premature neonates’ organs are not enough matured and one organ is likely to also affect the function of other organ, as a result of this the outcome of neonates was evolving and death.

The findings of this study indicate that the risk of neonatal death is significantly higher among neonates diagnosed with neonatal sepsis compared to those without sepsis. This may because of sepsis can rapidly progress in neonates, causing severe systemic inflammation and multi-organ failure, which can overwhelm their ability to cope. Moreover, the subtle symptoms of sepsis may be mistaken for other conditions, leading to delayed recognition and treatment, ultimately resulting in worse outcomes. Additionally, these findings align with existing literature that identifies neonatal sepsis as a significant contributor to neonatal mortality (34–36).

Moreover, the risk of neonatal death was significantly higher in neonates who had respiratory distress syndrome (*P* = 0.011) than neonates who haven't respiratory distress syndrome. This could be due to the fact that, respiratory distress significantly increases neonatal mortality due to factors like insufficient surfactant, leading to lung collapse, and impaired gas exchange. These issues can cause severe hypoxemia, acidosis, and multi-organ dysfunction, ultimately increasing the risk of death, especially in premature infants. The study was identified based on the documented data and could not display all factors that were not documented in the patient's files such as parents’ educational status, wealth index, training and educational status of health professional providing services, infection prevention practices. Thus, variations in physicians' clinical diagnosis, completeness and quality of the recorded information were limitations of the study.

## 5 Conclusion and recommendation

The study revealed that the magnitude of neonatal mortality was high which alarms the need of immediate collaborative actions for reduction of the burden particularly avoiding on the causal factors such as prematurity, sepsis and birth asphyxia which leads adverse birth outcomes. The majority of neonatal mortality was occurred in the first week of life. Therefore, maternal counseling, giving focused ante natal care as well as behavior change communications might be considered to promote positive behaviors are recommended to avoid the leading causes of neonatal mortality.

## Data Availability

The original contributions presented in the study are included in the article/Supplementary Material, further inquiries can be directed to the corresponding author.
